# Low Concentrations of Silver Nanoparticles Inhibit Spore Germination and Disturb Gender Differentiation of *Ceratopteris thalictroides* (L.) Brongn

**DOI:** 10.3390/nano12101730

**Published:** 2022-05-18

**Authors:** Zhenwei Lu, Liyan Yin, Wei Li, Hong-Sheng Jiang

**Affiliations:** 1 Hainan Key Laboratory for Sustainable Utilization of Tropical Bioresources, School of Life Sciences, Hainan University, Haikou 570228, China; luzhenwei599@163.com; 2 Jiangsu Coastal Area Institute of Agricultural Sciences, Yancheng 224002, China; 3 Key Laboratory of Aquatic Botany and Watershed Ecology, Wuhan Botanical Garden, Chinese Academy of Sciences, Wuhan 430074, China; liwei@wbgcas.cn; 4 One Health Institute, Hainan University, Haikou 570228, China

**Keywords:** silver nanoparticles, aquatic plants, *Ceratopteris thalictroides*, sex-dependent response, spore germination, aquatic fern

## Abstract

Because of their excellent antibacterial properties, silver nanoparticles (AgNPs) are widely used in all walks of life, which has caused them to be discharged into aquatic environments with possible negative effects on aquatic plants. In the present study, we used an aquatic fern, *Ceratopteris thalictroides*, as a model to investigate the effects of AgNPs on its spore germination, gametophytes, sex differentiation, and growth. The results demonstrated that AgNPs significantly inhibited spore germination of *C. thalictroides* at a AgNP concentration higher than 0.02 mg/L. Additionally, we found sex-dependent effects of AgNPs on the development and growth of the gametophyte of *C. thalictroides*. The proportion of hermaphrodites in the gametophytes and the area of gametophytes significantly decreased under AgNP treatment, while no significant effect was observed in the male gametophytes. Using the AgNP filtrate (without nanoparticles) and AgNPs plus cysteine (Ag^+^ chelator), we found that the release of Ag^+^ from nanoparticles was not the cause of the toxicity of AgNPs on *C. thalictroides*. The EC_50_ of AgNPs on spore germination was 0.0492 mg/L, thus indicating an ecological risk of AgNPs on this species even at concentrations lower than the Ag element concentration of the WHO guidelines for drinking-water quality.

## 1. Introduction

Due to their excellent antibacterial properties, silver nanoparticles (AgNPs) are widely used in various fields, including antibacterial coatings, medical machinery, cosmetics, clothing, food packaging, plastic products, detergent, paint, and some porous structures [1,2,3,4,5,6,7,8,9]. In the process of production, application, and recovery, AgNPs can be discharged into environments in many ways. According to a survey, about 60 tons of AgNPs are discharged into the aquatic environment each year [10]. Previous studies reported that the concentration of AgNPs in surface water in Europe was 0.5–2 ng/L, and the content of AgNPs in the final water of sewage-treatment plants was 32–111 ng/L. The content of AgNPs in activated sludge was 1.3–4.4 mg/kg [11,12]. The World Health Organization (WHO) guidelines for drinking-water quality state that the concentration of silver used to control bacteria in drinking water is 0.1 mg/L, which does not pose a risk to human health [13]. As the use of AgNPs increases, the content of AgNPs in water and sediment will increase, with potential negative effects to the ecosystems.

In recent years, an increasing number of studies have confirmed the toxicity of AgNPs to various organisms, including bacteria, algae, invertebrates, and higher plants, etc. [14,15,16,17]. The biological toxicity mechanism of AgNPs has been debated for decades, because it was not clear whether the toxicity comes from nanoparticles themselves or the release of Ag^+^ by AgNPs (Ag^+^_rel_). Previous studies considered that the Ag^+^_rel_ might be the main cause of the toxicity of AgNPs through the influence of AgNPs and AgNO_3_ on *Daphnia magna* and *Escherichia coli* [18,19]. Due to the small particle size and large specific surface area, the silver atoms are exposed to the outside of AgNPs, which leads to the release of more Ag^+^ into the environment. However, studies on *Lolium multiflorum* and *Spirodela polyrhiza* both indicated that the Ag^+^_rel_ may only be one reason for the toxicity of AgNPs [20,21]. Some studies showed that AgNPs absorbed by plants released Ag^+^ inside the cells, causing effects [22,23,24]. Recent studies have shown that the root system of wheat absorbed both the Ag^+^_rel_ and the AgNPs particles, resulting in toxic effects on wheat [25]. In vitro, Ag^+^_rel_ can interact with thiol groups in proteins, resulting in toxicity by enzyme inactivation or protein denaturation [26]. Although the extracellular oxidative release of Ag^+^ is the main cause of AgNPs toxicity in some cells and organisms, it still does not fully explain the toxicity mechanism of AgNPs [27,28,29,30]. Therefore, the study of the toxic mechanism of AgNPs requires further exploration. Additionally, at present, most of the studies on the phytotoxicology of AgNPs have focused on plant growth, but the research on their effects on plant reproduction remains limited. To date, only a few studies have reported that AgNPs reduce the germination rate and quality of *Arabidopsis thaliana* seeds [31]. AgNPs delay the bolting and flowering time of *A. thaliana* and affect its fruit and pod development [32].

*Ceratopteris thalictroides* (L.) Brongn is a pteridophyte that grows rooted or floating in shallow water, and is an endangered freshwater plant in China. *C. thalictroides* is one of the only vascular spore-bearing homosporous model species, which produce one type of spore, germinating into a gametophyte capable of producing both eggs and sperm. In the early stage of fern development, the environmental conditions to which spores are exposed can have a direct and vital impact on survival and reproduction. The main goal of the present study is to provide information on the phytotoxicity of AgNPs on pteridophytes. In particular, this study assesses the influence of environmentally relevant concentrations of AgNP on spore germination and the growth and development of *C. thalictroides*’ gametophytes. We hypothesized that: (1) Environmentally relevant concentrations of AgNPs could inhibit spore germination and disturb gender differentiation of *C. thalictroides*; (2) the particles themselves, but not Ag^+^_rel_, are mainly responsible for the toxicity of AgNPs.

## 2. Materials and Methods

### 2.1. Plant Material

**Figure 1:**
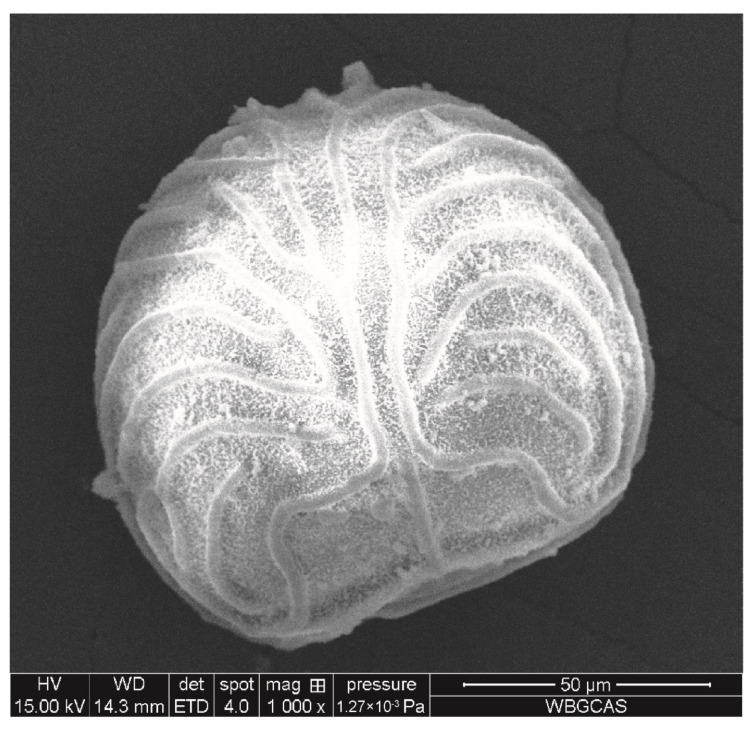
SEM image of spores of *Ceratopteris thalictroides*.

The spores of *C. thalictroides* were collected from plants in the Wuhan Botanical Garden, Chinese Academy of Sciences in November 2018 and stored at 4 °C, protected from light. The shape and size of spores were observed by a scanning electron microscope (SEM, DMi8, Leica, Germany). The specific process was as follows: spores with plump grains were randomly selected, repeated 5 times, and photos were taken. The software Image J (version 1.50) was used to measure spore size. The TEM image showed that the spore of *C. thalictroides* was nearly round or fan-shaped, and the spore surface has periodical ornamentation, which was formed by the uplift of the outer wall (Figure 1) and the spore diameter was 105.68 ± 3.83 μm (Table 1). 10 mg of spores were added into 10 mL distilled water, and the number of spores per mg was counted under microscopy. The density of spore was obtained as follows: approximately 1 mg spores were added into 1 mL distilled water, then a saturated sodium chloride solution was added drop by drop until the spores were completely suspended, 1 mL solution was weighted using a balance (SQP, with an accuracy of ±0.0001 g), and the density was calculated with three replicates. The spore density was 1.05 ± 0.04 g/cm^3^, the weight of individual spore was 0.41 ± 0.03 × 10^−6^ mg, and there were 2.45 ± 0.15 × 10^6^ spores per mg. (Table 1).

### 2.2. Characterization of AgNPs

The polyvinyl pyrrolidone (PVP) coating AgNPs stock (5030 mg/L) purchased from NanoComposix (San Diego, CA, USA) had an average diameter of 6.3 ± 1.4 nm and was stored at 4 °C, protected from light. To explore the effects of culture solution on AgNPs, the particle size and distribution of AgNPs in pure water and 10% Hoagland’s solution at 2.5 mg/L photomicrographs taken using a transmission electron microscope (TEM, HT-7700) were analyzed using Image J software. Additionally, the hydrodynamic diameter, polydispersity index (PDI), and Zeta potential of AgNPs at 5 mg/L were determined by using a surface potential analyzer (Dynamic Light Scattering, Nano ZS ZEN3600, Malvern, UK) in pure water and in 10% Hoagland’s solution. In the study, the PVP concentration of the coating substance of AgNPs was 2.85 mg/mL, and the Ag^+^_rel_ was determined as described in the previous study [26]. The measured Ag^+^_rel_ was 4.08 mg/L in 100 mg/L AgNPs suspension, meaning that AgNPs contained ~4.1% Ag^+^_rel_.

### 2.3. Effects of AgNPs on Spore Germination and Gametophyte Differentiation in C. thalictroides

AgNPs were diluted into 0, 0.02, 0.04, 0.06, 0.08, and 0.1 mg/L with 10% Hoagland’s solution in a step-by-step dilution method. AgNO_3_ was prepared by using 10% Hoagland’s solution with concentrations of 0, 0.02, 0.03, 0.04, 0.05, and 0.06 mg/L in a step-by-step dilution method. 5 mL AgNPs and AgNO_3_ solutions of each concentration were added to the 6-well cell-culture plate (Corning 3516, Corning NY, USA), and then approximately 100 spores were dropped into each well. Each treatment had 5 replicates. The cell-culture plates were incubated in a chamber under 16 h of light, 8 h of darkness, 25 ± 0.1 °C, and 20 μmol photons m^−2^ s^−1^ photosynthetically active radiation. After 8 days, the spore germination rate of *C. thalictroides* was observed and calculated under an electric stereoscopic fluorescence microscopy (SMZ25, Nikon Corporation, Tokyo, Japan). The production of false roots was used to assess spore germination. After that, the spore germination rate was observed every 2 days until the germination rate had not changed markedly. After 19 days, the numbers of hermaphrodites and male gametophytes were counted, and after 21 days, the area of the two kinds of gametophytes was counted. Concentration for 50% of maximal effect (EC_50_) of spore gemination rate at day 17 was calculated by a two-parameter logistic model with R software (R3.5.3 for Windows, R Foundation for Statistical Computing, Vienna, Austria).

### 2.4. Toxicity Sources of AgNPs on Spore Germination and Gametophyte Differentiation in C. thalictroides

To investigate whether the effect of AgNPs on spore germination was caused by particles or Ag^+^_rel_, the following 5 treatments were conducted: (1) 5 mg/L AgNPs were added into an ultrafiltration tube (0.5 mL, 30 KD, Millipore, Bedford, MA, USA) and centrifuged (3K15, Sigma, Osterode, Niedersachsen, Germany) at 8000 g at 4 °C for 10 min to obtain the AgNPs filtrate, which was diluted 62.5 times, which was equivalent to the concentration of 0.08 mg/L AgNPs filtrate; (2) 0.004 mg/L AgNO_3_ (AgNPs releases about 4.1% Ag^+^, which is about the same concentration as 0.08 mg/L AgNPs in the test); (3) 0.08 mg/LAgNPs + 0.09 mg/L cysteine (AgNPs + cys all, this concentration of cysteine can chelate all Ag^+^ even if all AgNPs is converted to Ag^+^); (4) 0.08 mg/L AgNPs + 0.0045 mg/L cysteine (AgNPs + cys part, this concentration of cysteine only can chelate the Ag^+^_rel_); (5) 0.09 mg/L cysteine (as the control, to determine whether cysteine affects spore germination of *C. thalictroides*). Approximately 5 mL of the solutions were added to the 6-well cell-culture plates, and then a drop of spore suspension, with about 100 spores of *C. thalictroides* per suspension drop, was added into each well. Each treatment was replicated five times. The plates were then incubated in a light chamber under 16 h of light, 8 h of darkness, 25 ± 0.1 °C, and 20 μmol photons m^−2^·s^−1^ photosynthetically active radiation. After 8 days of cultivation, the spore germination rate of *C. thalictroides* was observed under a stereomicroscope. The germination rate of *C. thalictroides* spores was observed every 2 days until the germination rate had not changed markedly. After 19 days, the number of hermaphrodites and male gametophytes was counted, and after 21 days, the area of the two gametophytes was counted.

### 2.5. Statistics Analysis

All data are presented as mean with one standard deviation. SPSS Statistics 20 was used for one-way ANOVA. If *p* < 0.05, the Tukey method was used to compare differences between groups. The particle size and potential of silver nanoparticles in pure water and culture medium were tested by independent sample *t*-test.

## 3. Results

### 3.1. Characterization of AgNPs

**Figure 2:**
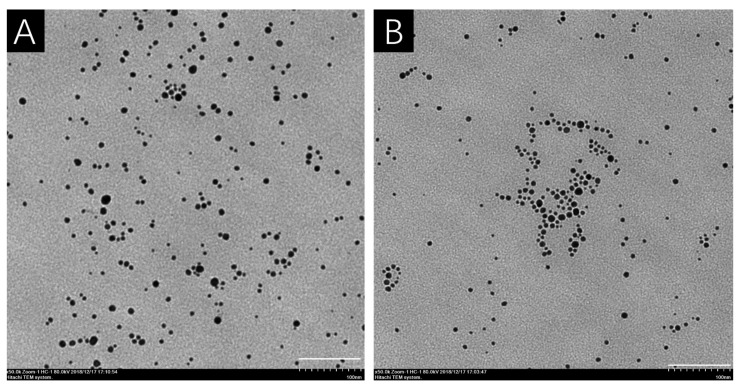
TEM image of AgNPs in distilled water (**A**) and 10% Hoagland’s solution (**B**). The white bars indicate 100 nm.

**Figure 3:**
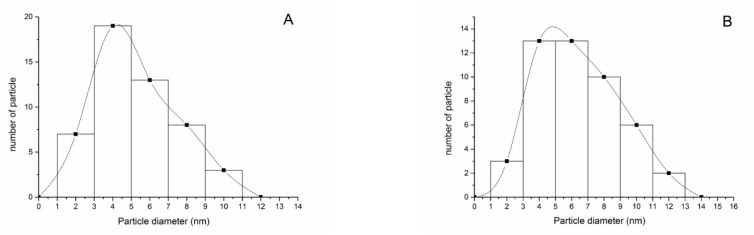
Size distribution of AgNPs in distilled water (**A**) and 10% Hoagland’s solution (**B**).

The shape of AgNPs was spherical under TEM both in distilled water and 10% Hoagland’s solution (Figure 2), and the measured core diameter was 6.2 ± 2.0 nm in distilled water and 7.8 ± 2.7 nm in 10% Hoagland’s solution, respectively (Table 2). From the TEM, most AgNPs were 4–6 nm in diameter both in distilled water and 10% Hoagland’s solution (Figure 3A,B). The DLS results showed that the hydrodynamic diameter of AgNPs increased from 20.3 nm in distilled water to 27.1 nm in 10% Hoagland’s solution (Table 2). Similarly, the DPI of AgNPs increased from 0.637 in distilled water to 0.791 in Hoagland’s solution (Table 2), while the Zeta potential of AgNPs in distilled water was −10.7 ± 0.4 mV which decreased significantly to −2.1 ± 0.4 mV in 10% Hoagland’s solution (Table 2).

### 3.2. Effects of AgNPs and Ag^+^ on the Spore Germination Rate of C. thalictroides

**Figure 4:**
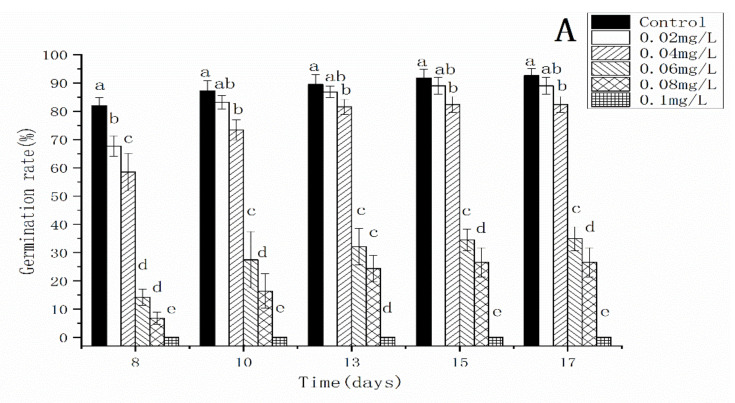

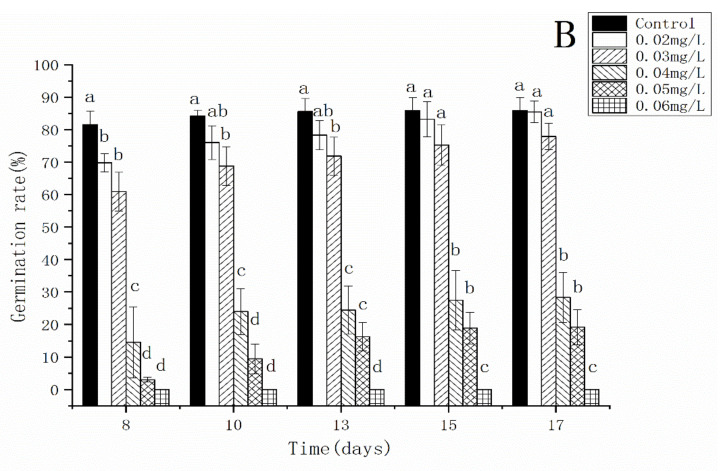
Effect of AgNPs (**A**) and Ag^+^ (**B**) on the spore germination of *Ceratopteris thalictroides*. Data represent the mean ± SD (*n* = 5). Data with different letters are significantly different (*p* < 0.05).

**Figure 5:**
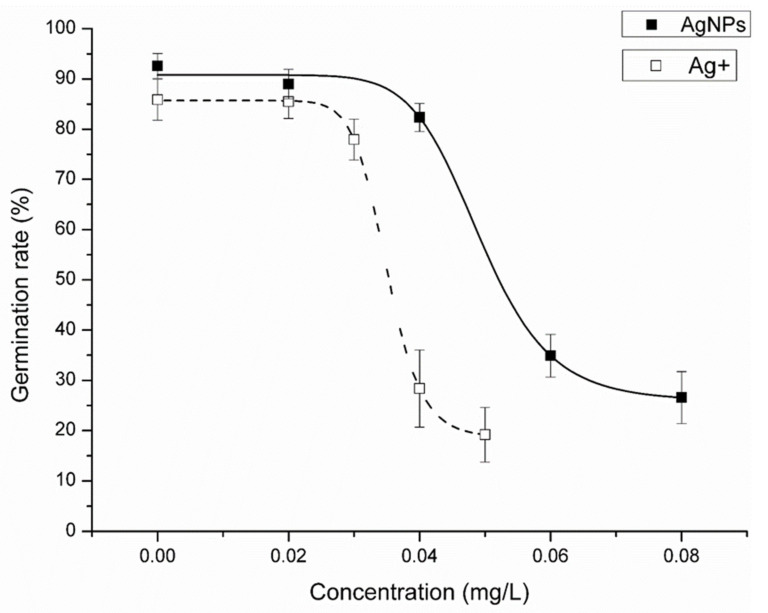
Concentration courses of effect of AgNPs and Ag^+^ on the spore germination rate of *Ceratopteris thalictroides* after 17 d exposure. Data represent the mean ± SD (*n* = 5).

In the control, more than 80% of spores had germinated by day 8. Although some spores continued to germinate after day 8, numbers were not significantly different between days 8 and day 17. The spore germination rate of *C. thalictroides* was significantly inhibited under AgNPs treatments and decreased significantly with increasing concentration of AgNPs (Figure 4A). Though 0.02 mg/L AgNPs did not significantly decrease the final germination rate, it delayed spore germination. When the concentration of AgNPs reached 0.06 mg/L, the spore germination rate was less than 30%. When the concentration of AgNPs reached 0.1 mg/L, no spore germination was observed. Ag^+^ showed a similar effect on spore germination as AgNPs (Figure 4B). After 17 days of exposure, the EC_50_ of AgNPs on spore germination was 0.0492 ± 0.0012 mg/L and the EC_50_ of Ag^+^ on spore germination was 0.0349 ± 0.0007 mg/L (Figure 5).

### 3.3. Effects of AgNPs and Ag^+^ on Gametophytes of C. thalictroides

**Figure 6:**
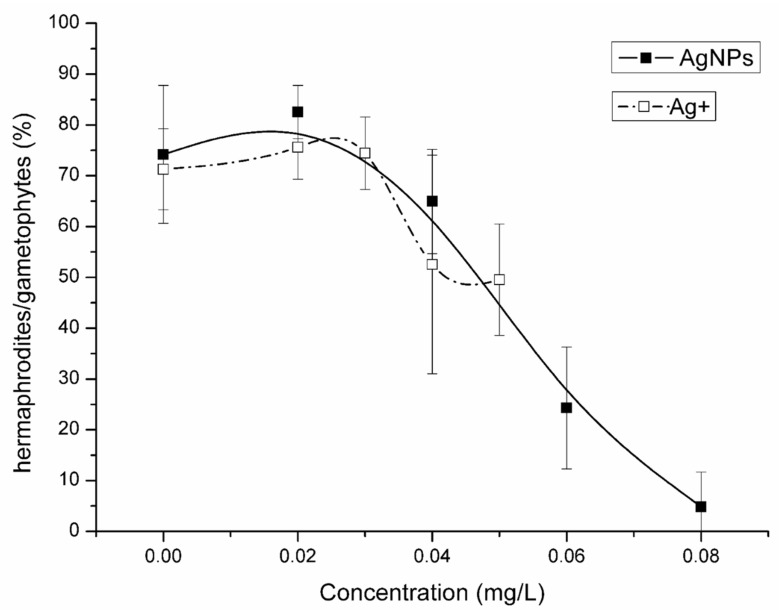
Effect of AgNPs and Ag^+^ on the proportion of hermaphrodites after 19 d exposure. Data represent the mean ± SD (*n* = 5).

After 19 days exposure to AgNPs, the proportion of hermaphrodite gametophytes decreased significantly with the increase in AgNP concentrations (Figure 6). When the AgNP concentration was at 0.02 and 0.04 mg/L, the proportion of hermaphrodites was 83% and 67%, which did not significantly differ from the control (75%). However, the hermaphrodites in the 0.06 and 0.08 mg/L AgNP treatments were 24% and 5% of the gametophytes, a decrease of 51% and 70% compared with the control. In comparison to the AgNP treatments, the hermaphrodites in the 0.05 mg/L Ag^+^ treatment group were 50% of the gametophytes, which decreased by 20% compared with the control group (Figure 6).

**Figure 7:**
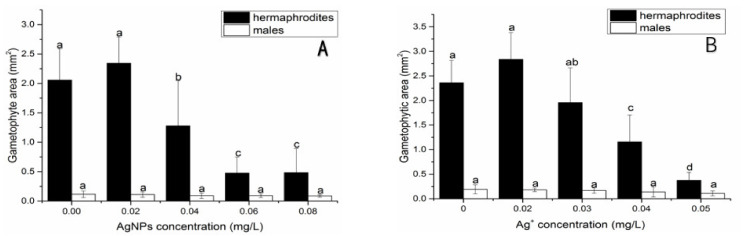
Effect of AgNPs (**A**) and Ag^+^ (**B**) on the area of male gametophytes and hermaphrodites of *Ceratopteris thalictroides* after 21 d exposure. Data represent the mean ± SD (*n* = 5). Data with different letters are significantly different among treatment within the same sexual gametophyte (*p* < 0.05).

After 21 days of exposure to AgNPs, the area of hermaphrodites decreased significantly with the increase in AgNPs concentration, while the area of male gametophytes did not change significantly (Figure 7A). When the concentration of AgNPs was 0.04 mg/L, the growth area of hermaphrodites decreased significantly compared with the control group, which was about 62.5% of it. When the concentration of AgNPs was 0.06 mg/L and 0.08 mg/L, the growth area of hermaphrodites was about 25% of that of the control. Similar to the AgNPs treatments, after 21 days of exposure to Ag^+^, the area of hermaphrodites decreased significantly with the increase of Ag^+^ concentration, while the area of male gametophytes did not change significantly (Figure 7B). When Ag^+^ concentration was 0.02 mg/L, no significant change was found in the area of hermaphrodites compared with the control group. When Ag^+^ concentration was 0.04 mg/L, the area of hermaphrodites decreased significantly compared with the control group, which was about 50% of it. When the concentration of Ag^+^ was 0.05 mg/L, the area of hermaphrodites was significantly reduced, which was about 16% of that of the control group.

## 4. Discussion

Previous studies have indicated that the toxicity of AgNPs was related to their particle size, and small particles were more toxic to organisms [9,20,33]. In our experiments, the core size of AgNPs did not change significantly, while the hydrodynamic diameter increased in 10% of Hoagland’s solution, indicating an AgNP aggregation in the solution. This aggregation was confirmed by the increase in PDI in our present study. Previous studies reported that cations in solution could be responsible for the aggregation of AgNPs by decreasing the zeta potential of nanoparticles, especially the divalent cation [26,34]. There were approximately 20 mg/L Ca^2+^, 5 mg/L Mg^2+^, 30 mg/L K^+^ and other trace metal ions in 10% Hoagland’s solution that could explain the aggregation of AgNPs. Though AgNPs aggregated in 10% of Hoagland’s solution, the size distribution showed that there were still some AgNPs with particle sizes of less than 5 nm. Dietz et al. reported that AgNPs with a particle size of 5 nm or smaller may penetrate plant cell walls and enter plant cells [35]. In this study, AgNPs with a particle size less than 5 nm may enter spores of *C. thalictroides* and interact with important bioactive substances such as proteins and/or nucleic acids in the cell, thus affecting spore germination and sex differentiation of gametophytes.

In the present study, the spore morphology and diameter of *C. thalictroides* were consistent with previous studies, with a diameter of 90–150 μm [36,37,38], indicating that the spores used in this study were mature spores with normal development. Our results showed that AgNPs or Ag^+^ significantly inhibited spore germination of *C. thalictroides*, and the EC_50_ of AgNPs and Ag^+^ on *C. thalictroides* spore germination was 0.0492 ± 0.0012 mg/L and 0.0349 ± 0.0007 mg/L, respectively. According to the World Health Organization (WHO) guidelines, the concentration of silver used to control bacteria in drinking water is 0.1 mg/L [13]. In our study, 0.1 mg/L AgNPs or Ag^+^ can significantly inhibit the spore germination of *C. thalictroides* to achieve bacteriostatic purposes. Spore germination is crucial in the life history of *C. thalictroides*. Even if the Ag element in the water was within the recommended range of drinking-water guidelines, it could significantly inhibit the spore germination of *C. thalictroides*, which may have a significant impact on their reproduction. Many studies have shown that AgNPs had toxic effects on animals, higher plants, bacteria, and fungi [39,40,41,42,43,44,45,46,47,48,49]; however, the concentration used was rather high, and the EC_50_ of AgNPs was usually more than 1 mg/L. *Ceratopteris thalictroides* is one of the most sensitive species to the toxicity of AgNPs or Ag^+^, and thus could be used as a Ag-toxicity indicator.

In this study, AgNPs significantly affected the sex differentiation of gametophytes of *C. thalictroides*. AgNPs significantly inhibited the differentiation of gametophytes into hermaphrodites, and at a concentration of 0.08 mg/L the proportion of hermaphrodites decreased by 70% compared with the control. In contrast, AgNPs had no significant effect on the differentiation into male gametophytes (Figure S1A). These results indicate that the sensitivity of gametophytes from *C. thalictroides* to AgNPs was sex-dependent. A previous study reported that delayed spore germination of *C. thalictroides* could affect the sex differentiation of *C. thalictroides*’ gametophytes [50], which could explanation our sex-dependent result. However, the effect of Ag^+^ on sex differentiation of gametophytes was different from that of AgNPs. Although Ag^+^ significantly decreased the proportion of hermaphrodites as AgNPs, both numbers of hermaphrodites and male gametophytes decreased; hermaphrodites decreased faster than males and resulted in a decrease in the proportion of hermaphrodites (Figure S1B). Previous studies showed that 0.1 mg /L microplastics resulted in a 30% decrease in the proportion of hermaphrodites in *C. thalictroides* compared to the control group and that 4 mg/L quinclorac resulted in a 30% decrease in the proportion of hermaphrodites compared with the control group [51,52]. Compared with Ag^+^, the traditional herbicide quinclorac, and new pollutant microplastics, AgNPs had a greater effect on the sex differentiation of gametophytes of *C. thalictroides*. The sex-dependent response also showed in the growth of gametophytes. In this study, the area of gametophytes of different genders responded differently to the toxicity of AgNPs. The area of hermaphroditic gametophytes induced by AgNPs at 0.08 mg/L was 25% of that in the control group. The results of this experiment showed that AgNPs significantly reduced the area of hermaphrodites, which may lead to a decrease in the biomass of *C. thalictroides*, affecting its normal growth, development, and breeding populations [53]. Ag^+^ significantly affected the area of gametophytes of *C. thalictroides*; the area of hermaphrodites exposed to 0.05 mg/L Ag^+^ was 16% that of control hermaphrodites. However, the area of male gametophytes in the 0.08 mg/L AgNP treatment and the 0.05 mg/L Ag^+^ treatment was not significantly different from that in the control. Different gender sensitivity to the toxicity of AgNPs has also been observed in animals. Studies showed that AgNPs accumulated in the ovaries, resulting in abnormal follicular development of female fish, resulting in the loss of their reproductive ability, and also resulting in embryonic dysplasia of female fish, reducing the survival rate of embryos [53,54,55,56,57,58,59]. When AgNPs interacted with serum proteins of *Micropterus dolomieu*, the formation of protein corona of AgNPs was sex-dependent, mainly because female serum contained some egg-specific proteins such as vitellogenin and zona pellucida, etc. This may lead to differences in the response of female and male smallmouth bass (*Micropterus dolomieu*) to AgNPs toxicity [60]. The sex-dependent response of *C. thalictroides*’ gametophytes differentiation to AgNPs may be caused by the differential expression of sex-specific proteins during the process of gametophyte differentiation.

It is a hot topic in the toxicity of AgNPs whether it depends on Ag^+^_rel_ or the particle itself. In the present study, the EC50 of AgNPs and Ag^+^ on spore germination by *C. thalictroides* was 0.0492 ± 0.0012 mg/L and 0.0349 ± 0.0007 mg/L, respectively, which indicated that Ag^+^ is more toxic than AgNPs, but the toxicity was in the same order of magnitude. Thus, the low concentration of Ag^+^_rel_ could not explain the high toxicity of AgNPs. In the present study, the effects of the 0.08 mg/L AgNP filtrate treatment on spore germination (Figure S2), sex differentiation of gametophytes (Figure S3), and area of gametophytes (Figure S4) were not significantly different from those of the control group, and they significantly differed from the 0.08 mg/L AgNP treatment. About 4.1% Ag^+^ was released by 0.08 mg/L AgNPs, so about 0.004 mg/L Ag^+^ was released in the 0.08 mg/L treatment. The effects of 0.004 mg/L Ag^+^ treatment on spore germination, sex differentiation of gametophytes, and area of gametophytes were not significantly different from those of the control. In addition, 0.004 mg/L Ag^+^ was much lower than the EC50 of Ag^+^ on spores of *C. thalictroides*. These results indicate that the Ag^+^ released by AgNPs did not have a significant effect on the germination and growth of spores of *C. thalictroides*, and the toxicity of AgNPs may not be caused by the Ag^+^_rel_. The effects of 0.08 mg/L AgNPs + 0.09 mg/L cysteine, 0.08 mg/L AgNPs + 0.045 mg/L cysteine and 0.09 mg/L cysteine on spore germination, sex differentiation of gametophytes, and area of gametophytes were not significantly different from those of the control group. The results showed that when cysteine was mixed with Ag^+^, AgNPs had no significant effect on spore germination, sex differentiation of gametophytes, and growth area of gametophytes, suggesting that the toxicity of AgNPs to the spores of *C. thalictroides* might be changed by cysteine. AgNPs interacting with organic matter in solution should be considered in its toxicity. When the cysteine was combined with the Ag^+^_rel_ or AgNPs itself, it formed a passivation layer on the surface of AgNPs. This prevents the further release of Ag^+^ and also prevents interaction between AgNP particles and spores of *C. thalictroides* (Figures S2–S4). Some studies have shown that Cl^−^ can react with dissolved Ag^+^ to generate a silver chloride passivation layer on AgNPs, reducing the toxicity of AgNPs [61,62,63]. Additionally, in the present study, the cysteine as a chelator of Ag^+^ could also be one of the antioxidants [64] that had a possibility to alleviate the toxicity of AgNPs on *C. thalictroides*. In conclusion, the Ag^+^_rel_ cannot explain the highly toxic effect of AgNPs on the spores of *C. thalictroides*. The interaction among AgNPs, material in solution, and the spores of *C. thalictroides* could be important in the toxic mechanisms of AgNPs. Thirty percent of the AgNPs used in this study had a particle size of less than 5 nm, which could be likely to enter the spores of *C. thalictroides* and produce effects. Previous studies have shown that AgNPs entered animal and plant cells where they released Ag^+^, thus producing toxic effects [23,24,25,26]. This is a further direction for our work on the toxic effects of AgNPs on spores of *C. thalictroides*.

## 5. Conclusions

In this study, it was found that AgNPs significantly inhibited the spore germination of *C. thalictroides*. Moreover, AgNPs also significantly affected further gametophyte development, and these effects were sex-dependent. AgNPs significantly inhibited the development of hermaphrodites and significantly inhibited their growth. According to the World Health Organization (WHO) guidelines, the concentration of silver used to control bacteria in drinking water is 0.1 mg/L. In our study, 0.1 mg/L AgNPs or Ag^+^ can completely inhibit the spore germination of *C. thalictroides*.

By comparing the toxicity of AgNPs and Ag^+^, it was found that the toxicity of AgNPs to *C. thalictroides* was slightly lower than that of Ag^+^, but the toxicity of the two forms of silver was in the same order of magnitude. It was found that the Ag^+^_rel_ was not the main source of the toxicity of the AgNPs.

The sensitivity of spore of *C. thalictroides*’ to AgNPs was greater than in other studied higher plants, animals, and even some bacteria, fungi, algae. It was particularly sensitive to the toxicity of AgNPs, implying that the real concentration of AgNPs in some aquatic environments may affect spore germination of *C. thalictroides* which may affect its reproduction. Since *C. thalictroides* is one of the most sensitive species to the toxic effects of AgNPs or Ag^+^, it could be used as an Ag toxicity indicator.

## Figures and Tables

**Table 1:** Diameter, density, and weight of *Ceratopteris thalictroides*. Data represent the mean ± SD (*n* = 5).

Diameter (μm)	Density (g/cm^3^)	Weight (mg)	Number of Spores per Milligram
105.68 ± 3.83	1.05 ± 0.04	0.41 ± 0.03 × 10^−6^	2.45 ± 0.15 × 10^6^

**Table 2:** Core diameter, hydrodynamic diameter, polydispersity index (PDI) and Zeta potential of AgNPs in distilled water (A) and 10% Hoagland’s solution (B). Data represent the mean ± SD (*n* = 5). Data with different letters are significantly different (*p* < 0.05).

Solution	Core Diameter * (nm)	Hydrodynamic Diameter * (nm)	PDI	Zeta Potential (mV)
H_2_O	6.2 ± 2.0	20.3 ± 3.1	0.637	−10.7 ± 0.4 a
10% Hoagland’s solution	7.8 ± 2.7	27.1 ± 4.1	0.791	−2.1 ± 0.4 b

* Core diameter was obtained by using a TEM; hydrodynamic diameter was obtained by using dynamic light scatter.

## Data Availability

Not applicable.

## Supplementary Materials

The following supporting information can be downloaded at: https://www.mdpi.com/article/10.3390/nano12101730/s1, Figure S1: Effect of AgNPs (A) and Ag^+^ (B) on the number of gametophytes of *Ceratopteris thalictroides* after 19 d exposure. Data represent the mean ± SD (*n* = 5). Data with different letters are significantly different (*p* < 0.05); Figure S2: Effect of various comparison groups on the spore germination of *Ceratopteris thalictroides* after 17 d exposure. (Control; AgNPs filtrate: 0.08 mg/L AgNP filtrate; Ag^+^: 0.004 mg/L Ag^+^; AgNPs + cys(all): 0.08 mg/L AgNPs + 0.09 mg/L cys; AgNPs + cys(part): 0.08 mg/L AgNPs + 0.0045 mg/L cys; Cys: 0.09 mg/L cys) Data represent the mean ± SD (*n* = 5). Data with same letters are not significantly different (*p* = 0.05); Figure S3: The proportion of hermaphrodites of various comparison groups after 19 d exposure. (Control; AgNPs filtrate: 0.08 mg/L AgNP filtrate; Ag^+^: 0.004 mg/L Ag^+^; AgNPs + cys(all): 0.08 mg/L AgNPs + 0.09 mg/L cys; Cys: 0.09 mg/L cys; AgNPs + cys(part): 0.08 mg/L AgNPs +0.0045 mg/L cys) Data represent the mean ± SD (*n* = 5). Data with same letters are not significantly different (*p* = 0.05); Figure S4: Effect of various comparison groups on area of male gametophytes and hermaphrodites of *Ceratopteris thalictroides* after 21 d exposure. (Control; AgNP filtrate: 0.08 mg/L AgNP filtrate; Ag^+^: 0.004 mg/L Ag^+^; AgNPs + cys(all): 0.08 mg/L AgNPs +0.09 mg/L cys; Cys: 0.09 mg/L cys; AgNPs + cys(part): 0.08 mg/L AgNPs + 0.0045 mg/L cys) Data represent the mean ± SD (*n* = 5). Data with same letters are not significantly different (*p* = 0.05).
