# Supplementary material for: Low Concentrations of Silver Nanoparticles Inhibit Spore Germination and Disturb Gender Differentiation of Ceratopteris thalictroides (L.) Brongn
Source: Nanomaterials (Basel). 2022 May 18;12(10):1730. doi: 10.3390/nano12101730 (PMC9143685; doi:10.3390/nano12101730)
Supplement: Supplementary file 1 [file nanomaterials-12-01730-s001.zip › nanomaterials-1681431-supplementary.pdf]

nanomaterials

Supplementary Materials

Low Concentrations of silver Nanoparticles Inhibit Spore Germination and Disturb Gender Differentiation of *Ceratopteris thalictroides* (L.) Brongn

Zhenwei Lu [1,2,3], Liyan Yin [1,4,*], Wei Li [3] and Hong-Sheng Jiang [3,*]

1 Hainan Key Laboratory for Sustainable Utilization of Tropical Bioresources, School of Life Sciences, Hainan University, Haikou 570228, China; luzhenwei599@163.com
2 Jiangsu Coastal Area Institute of Agricultural Sciences, Yancheng 224002, China
3 Key Laboratory of Aquatic Botany and Watershed Ecology, Wuhan Botanical Garden, Chinese Academy of Sciences, Wuhan 430074, China; liwei@wbgcas.cn
4 One Health Institute, Hainan University, Haikou 570228, China
* Correspondence: lyyin@163.com (L.Y.); jhs@wbgcas.cn (H.S.J.); Tel: +86-898-6616-0721 (L.Y.); Tel: +86-27-8770-0855 (H.S.J.)

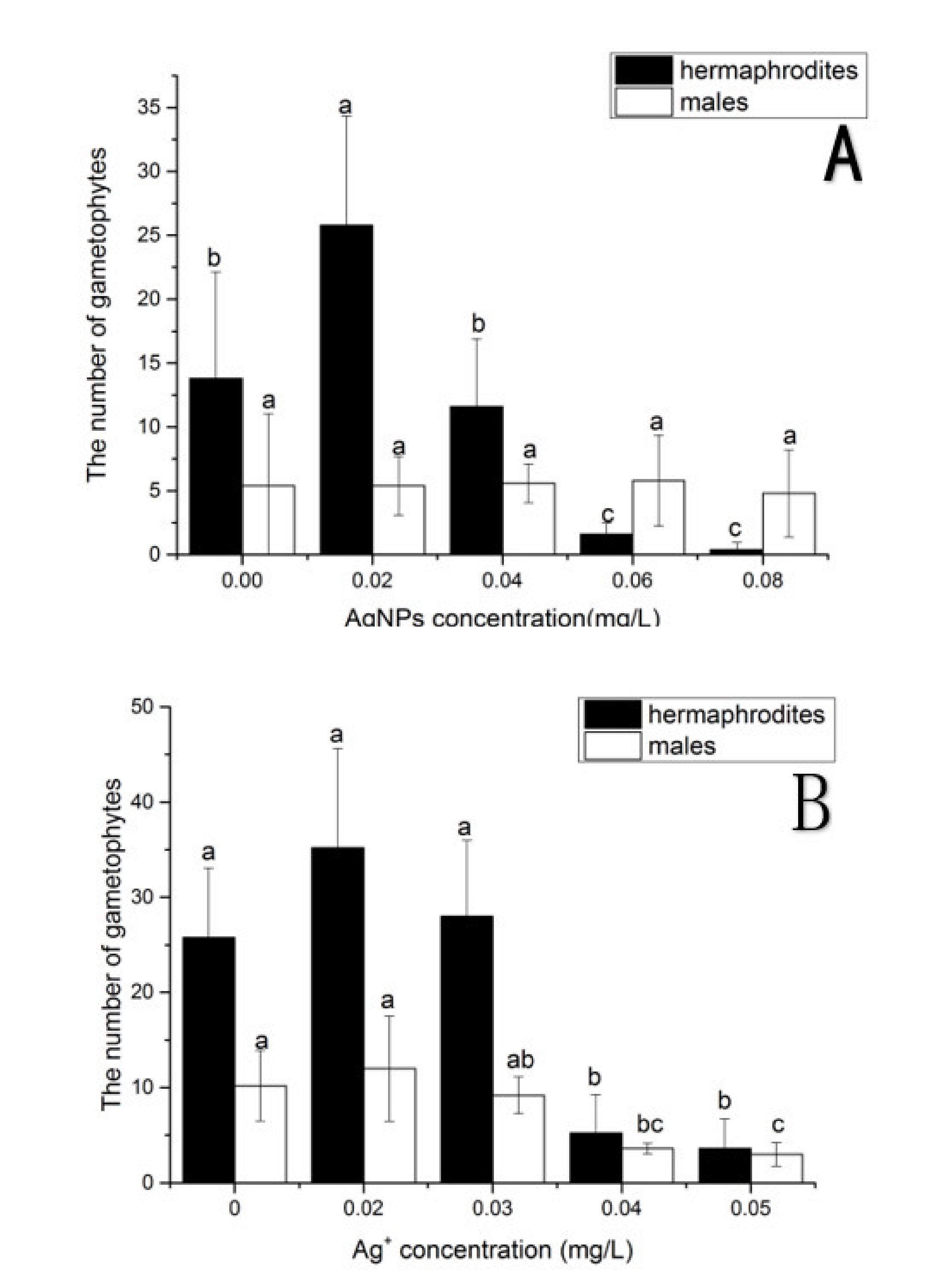

Figure S1. Effect of AgNPs (**A**) and Ag^{+} (**B**) on the number of gametophytes of *Ceratopteris thalictroides* after 19d exposure. Data represent the mean ± SD (n = 5). Data with different letters are significantly different ($p < 0.05$).

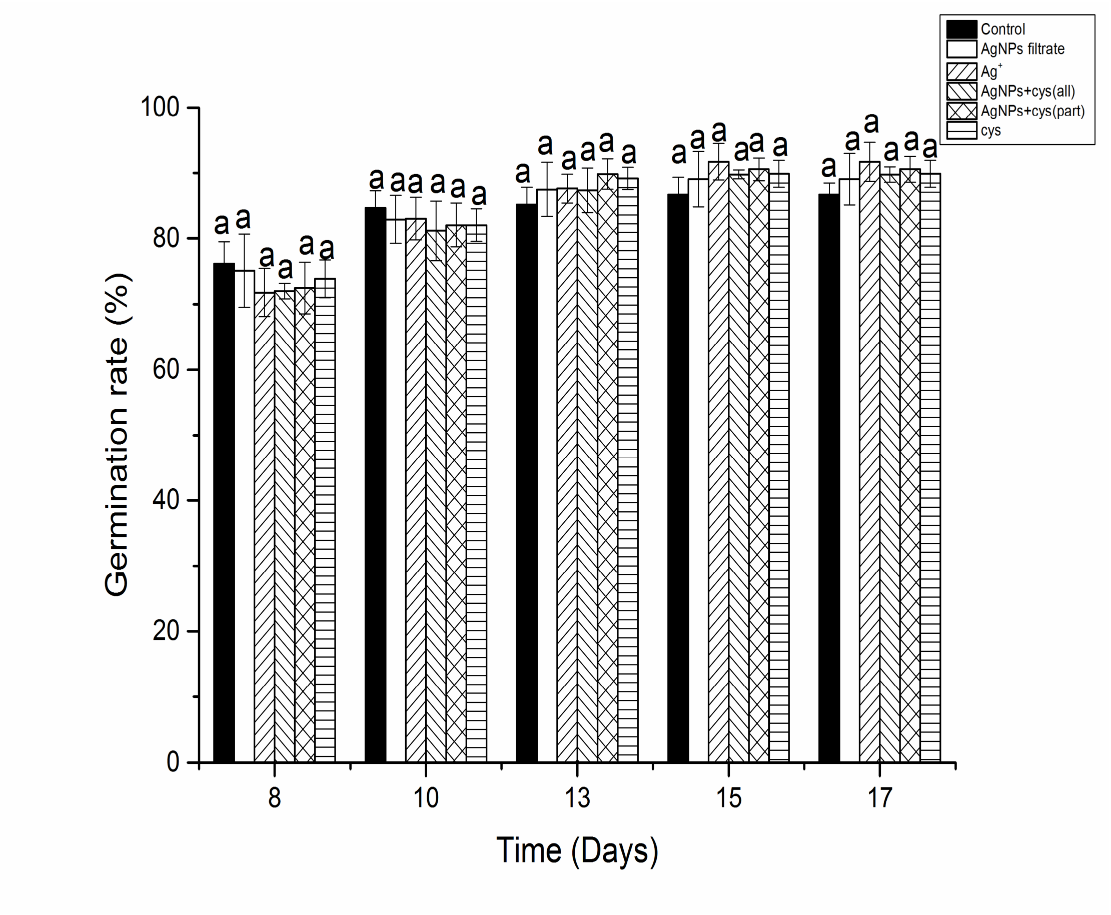

Figure S2. Effect of various comparison groups on the spore germination of *Ceratopteris thalictroides* after 17d exposure. (Control; AgNPs filtrate: 0.08 mg/L AgNPs filtrate; Ag^+: 0.004 mg/L Ag^+; AgNPs + cys(all): 0.08 mg/L AgNPs + 0.09 mg/L cys; AgNPs + cys(part): 0.08 mg/L AgNPs + 0.0045 mg/L cys; Cys: 0.09 mg/L cys) Data represent the mean ± SD (n = 5). Data with same letters were not significantly different (p = 0.05).

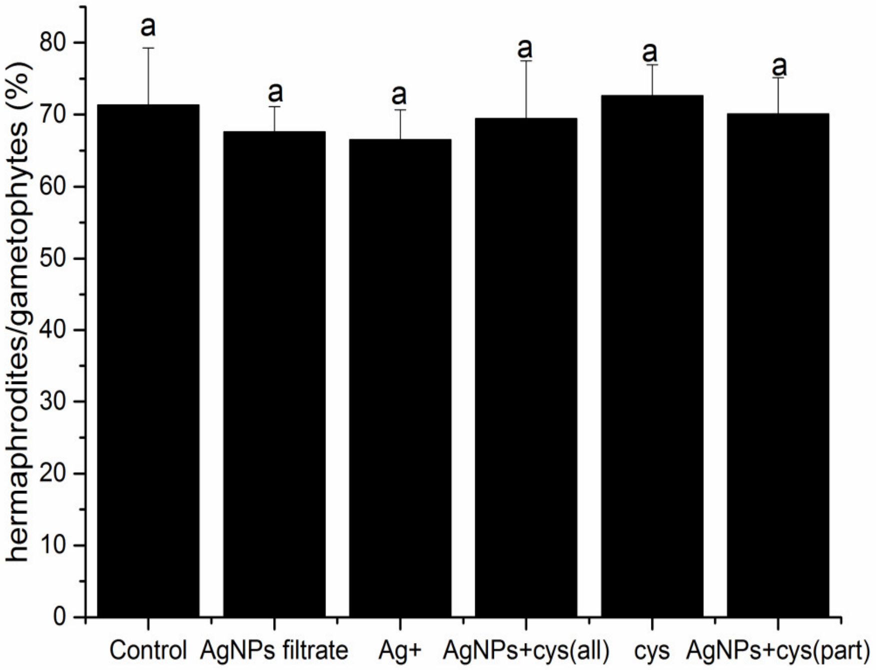

Figure S3. The proportion of hermaphrodites of various comparison groups after 19d exposure. (Control; AgNPs filtrate: 0.08 mg/L AgNPs filtrate; Ag^+: 0.004 mg/L Ag^+; AgNPs + cys(all): 0.08 mg/L AgNPs + 0.09 mg/L cys; Cys: 0.09 mg/L cys; AgNPs + cys(part): 0.08 mg/L AgNPs +0.0045 mg/L cys) Data represent the mean ± SD (n = 5). Data with same letters were not significantly different (p = 0.05).

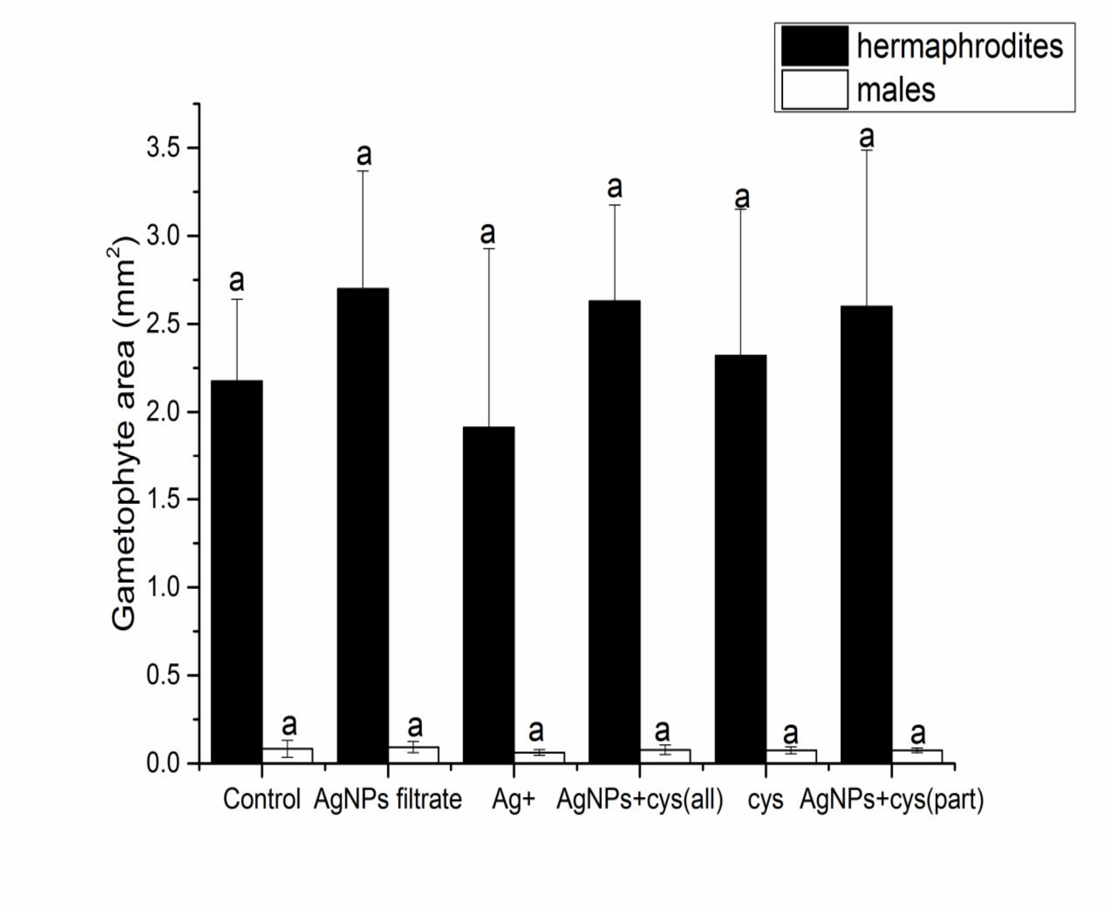

Figure S4. Effect of various comparison groups on area of male gametophytes and hermaphrodites of *Ceratopteris thalictroides* after 21d exposure. (Control; AgNPs filtrate: 0.08 mg/L AgNPs filtrate; Ag^{+}: 0.004 mg/L Ag^{+}; AgNPs + cys(all): 0.08 mg/L AgNPs +0.09 mg/L cys; Cys: 0.09 mg/L cys; AgNPs + cys(part): 0.08 mg/L AgNPs + 0.0045 mg/L cys) Data represent the mean ± SD ($n = 5$). Data with same letters were not significantly different ($p = 0.05$).
